# Respiratory virus deterrence induced by modified mask filter

**DOI:** 10.1371/journal.pone.0257827

**Published:** 2021-09-30

**Authors:** Su-Hwa Lee, Ki-Back Chu, Hae-Ji Kang, Min-Ju Kim, Eun-Kyung Moon, Fu-Shi Quan

**Affiliations:** 1 Department of Medical Zoology, Kyung Hee University School of Medicine, Seoul, Republic of Korea; 2 Department of Biomedical Science, Graduate School, Kyung Hee University, Seoul, Republic of Korea; 3 Department of Medical Research Center for Bioreaction to Reactive Oxygen Species and Biomedical Science Institute, School of Medicine, Graduate school, Kyung Hee University, Seoul, Republic of Korea; VIT University, INDIA

## Abstract

Airborne transmission of infectious respiratory pathogens is a significant health hazard for the general public as well as healthcare professionals. Face masks have been frequently utilized as safety measures to limit the transmission of these infectious aerosolized particles. However, the efficacy of face masks in reducing respiratory virus infectivity and pathogenicity is unknown. Improving the effectiveness of masks in blocking viruses is urgently needed. In this study, surgical mask filters were modified by coating the filters with 1, 3, or 5 M of sodium dihydrogen phosphate, and subsequently exposed to the aerosolized respiratory influenza viruses (A/H3N2, A/H5N1) generated by a nebulizer set. Mask filter modification significantly reduced the size and counts of filter pores, which enabled entrapment of 40–60% of aerosolized viruses (captured viruses) with more than 90% of the captured viruses losing their infectivity. Upon contact with the coated mask filters, both the captured viruses and the viruses that managed to bypass the filter pore (passed viruses) were found to be inactivated. Passed viruses demonstrated significantly reduced pathogenicity in mice as indicated by significantly reduced lung virus titers, bodyweight loss, and prolonged survival compared to bare control. These findings highlight the potential of modified mask filters for reducing viral activity and pathogenicity, which contributes to improving facial mask efficacy as well as limiting airborne pathogen transmission.

## Introduction

Aerosols are airborne particles composed of both liquid and solid components which can be generated through physicochemical reactions of various substances, including infectious pathogens such as bacteria, fungi, and viruses [1]. Airborne transmission via aerosol is one of the major modes of transmitting respiratory diseases between humans, with the others being physical contact through fomites and transmission of pathogen-laden droplets to the mucosal surfaces of individuals through forceful expulsions [1]. The classification of these respiratory particles as either aerosol or droplet depends on their size, with the droplet being the larger of the two [2]. Under closed environmental conditions, aerosol particles encompassing infectious pathogens can remain suspended in the air almost indefinitely unless external intervention is introduced [3,4]. One of the key aspect determining the aerodynamic behavior of the aerosol particles is their size, which seems to influence the aerosol propensity to remain suspended in air, deposition sites in humans, and their potential to initiate an infection in the respiratory tract [5]. While individuals exposed to these infectious aerosols are at imminent risk of infection, equipping surgical masks or N95 respirators can thwart the airborne transmission of infectious microbial pathogens such as *Bordatella pertussis* and Ebola virus [3,6].

Several studies have reported conflicting findings regarding the protection conferred through surgical masks and N95 respirator usage. Although surgical masks can prevent hand-to-face contact and facial contact with respiratory droplets, it does not prevent the wearer from inhaling small airborne particles [7]. N95 respirators worn by healthcare personnel prevents small airborne particle inhalation and fits tightly to the wearer’s face, thereby limiting facial seal leakage [8]. Surprisingly, a study reported that the effectiveness of N95 respirators was not significantly different from medical masks for preventing influenza or other respiratory diseases [9]. Another study reported that surgical face masks could prevent the transmission of human coronaviruses and influenza viruses from symptomatic individuals [10]. However, surgical masks cannot efficaciously reduce the emission of influenza virus particles into the environment in aerosols [10]. The controversy over whether various masks are effective in protecting people from respiratory virus infections needs to be clarified. More importantly, mask filter modification that can prevent respiratory viruses is urgently needed.

Recently, it was reported that aerosolized influenza virus subtypes A/H1N1 and A/H5N1 captured on sodium chloride-coated mask filters underwent rapid infectivity loss compared to controls, with the viruses being killed during the salt recrystallization process [11]. Low cost and safety are two aspects of sodium chloride that may signify its potential applicability to mask filters, but mask wearers can be subjected to olfactory and gustatory discomfort from prolonged salt exposure. Furthermore, since the amount of respiratory virus particles in the aerosols can vary over time, accurate measurement of virus particles in the aerosol bypassing the mask filters remain unknown. As such, continuous efforts to improve the respiratory pathogen-blocking efficacy of masks using a better salt is urgently needed.

Thus, in the current study, sodium dihydrogen phosphate (pH 4.0) which was approved for consumption by the U.S. Food and Drug Administration (FDA) was selected. Both sodium chloride and sodium dihydrogen phosphate are cheap but unlike the former, sodium dihydrogen phosphate is odorless and tasteless. Mask filters were coated with various concentrations of sodium dihydrogen phosphate, and influenza viruses aerosolized at high and low concentrations were exposed to the salt-coated mask filters to assess virus filtration efficiency and virus infectivity loss *in vitro* and *in vivo*. Findings of the present study indicate that salt-coated filters significantly reduced virus infectivity, which could greatly contribute to the prevention of respiratory pathogen transmission.

## Materials and methods

### Ethics statement

All animal studies and husbandry involved in these experiments were conducted by following the guidelines established by Kyung Hee University IACUC, which operates under the National Veterinary Research and Quarantine Service (NVRQS) and regulations of the World Organization for Animal Health (WOAH). All experimental procedures involving animals were reviewed, approved, and supervised by the institutional animal research ethics committee (permit number: KHSASP-19-188). All researchers were trained in animal care and handling. They received the certificate of completion for the Animal Welfare & Ethics Course from CITI. Every effort was made to minimize animal suffering.

### Preparation of live influenza virus

Influenza viruses A/Hong Kong/1/1968 (H3N2) or A/Viet Nam/1203/04 (H5N1) were grown in 10-day-old embryonated hen’s eggs for 2.5 days. The influenza virus in the allantoic fluid of infected eggs was harvested after cold room incubation overnight and concentrated from the allantoic fluid by high-speed centrifugation at 30000 rpm for 1h. Influenza viruses were purified using a discontinuous sucrose gradient (15%, 30%, and 60%) layers, and viruses were pelleted by high-speed centrifugation at 30000 rpm for 1h. Sedimented pellets were resuspended in 500 μl of 0.1 M phosphate-buffered saline (PBS) and incubated at 4°C overnight. Viruses were stored at -80°C until used.

### Preparation of salt-coated filters

The commercial surgical masks had three layers. The inner and outer layers of the mask serve to protect the filter from detrition. To obtain a middle layer (polypropylene filter) of the mask, a commercially available mask (Guardman 3 Ply disposable band mask; Dainnuri Co., Incheon, Korea) was cut to remove the inner and outer layers, and the middle layer was processed into a circular size of 85 mm in diameter. Sodium dihydrogen phosphate coating solutions were dissolved (NaH_2_PO_4_, Sigma Aldrich, St. Louis, MO) in deionized (DI) water by stirring at 400 rpm at 80°C, followed by the addition of Tween 20 (Sigma Aldrich, St. Louis, MO) to a final concentration of 1M, 3M or 5M of NaH_2_PO_4_ and 1% of Tween 20. To obtain the salt-coated filters, the mask filters were immersed in 1 ml of coating solution on 90 ⅹ 15 mm petri dish (SPL Life Sciences, Gyeonggi-do, Korea) and incubated overnight at room temperature. Subsequently, the filters were dried in an oven (KB1250 incubator; K&K Scientific Supplier, Seoul, Korea) at 37°C for 24 h. The dried non-coating filters (bare) and salt-coated filters (1M, 3M, and 5M) were finally prepared by cutting into a circular shape having a diameter of 25mm.

### Scanning electron microscopy (SEM) and intake resistance

To identify the pore size and pore counts of mask filters, SEM analysis was performed for non-coated filter and salt-coated filters. SEM analysis (S-4700; Hitachi, Tokyo, Japan) was operated in secondary electron mode at 10kV. To investigate the breathability of filters, intake resistance of mask filters was confirmed by Certitest ® Automated Filter Tester (TSI8130A-EN, TSI incorporated, Minnesota, USA) at Wooilcntech company (Pyeongtaek, Korea).

### Virus exposure to filters

**Fig 1:**
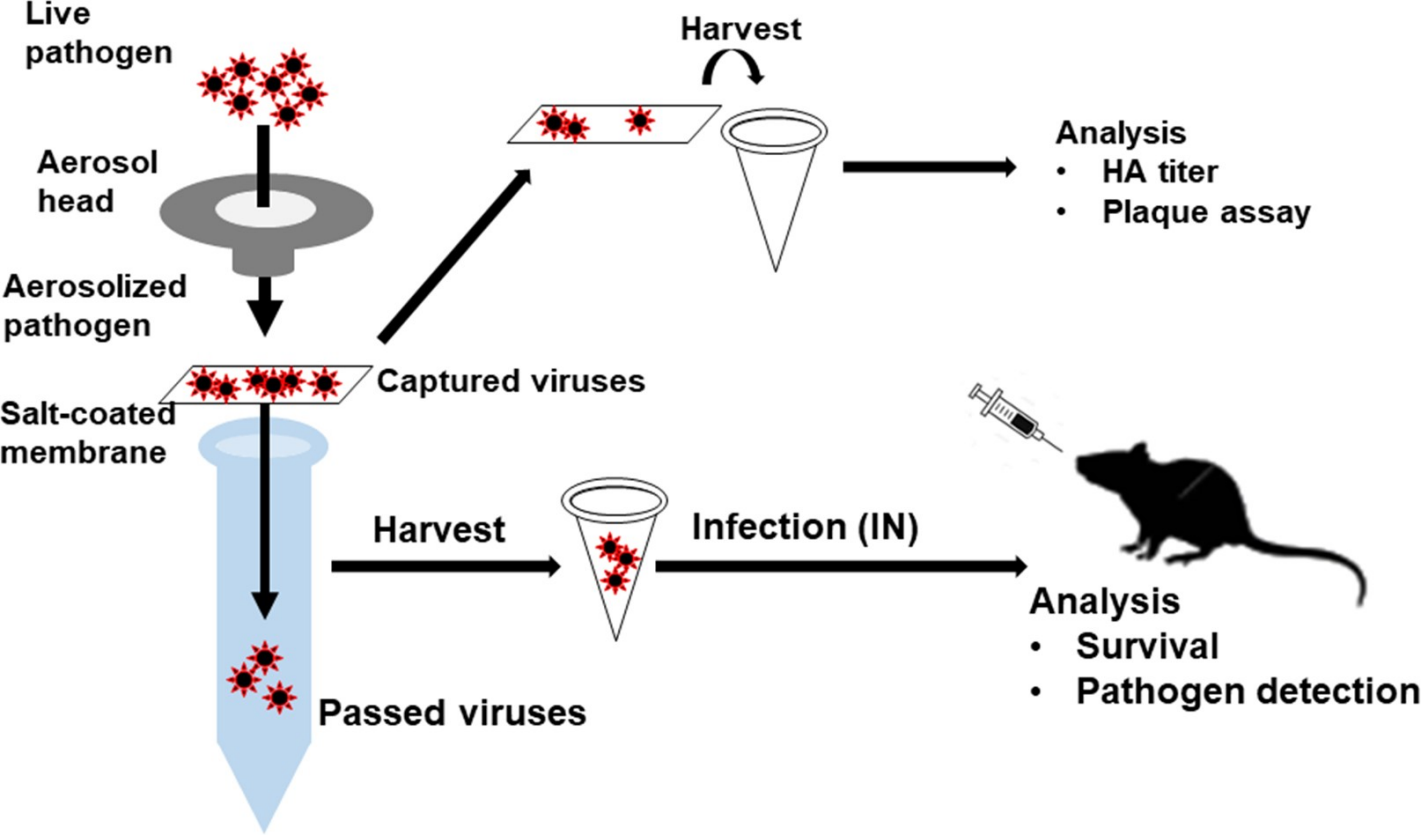
Experimental design. Live influenza viruses (A/H3N2, A/H5N1) were aerosolized by aerosol generator nebulizer set. Virus aerosols were exposed to the surgical mask filters, and the viruses either captured or passing through filters were harvested. Both captured and passed viruses were analyzed for virus hemagglutinin activity, infectivity, and pathogenicity in mice.

Influenza viruses A/H3N2 (8 mg/ml and 2 mg/ml) and A/H5N1 (2 mg/ml) were aerosolized using an aerosol generator device. Conical tubes (15ml) filled with 1 ml of DI water was tightly inserted into the cylindrical nebulizer unit (aerosol head, diameter: 20 mm, height: 20 mm) of aerosol generator in nebulizer set (Aeroneb Lab Nebulizer system; Aerogen, Galway, Ireland). The mask filter was placed between the cylindrical nebulizer unit and the conical tube (Fig 1). Ten microliters of virus sample were added to the nebulizer unit and aerosols (VMD 2.5–4 μm from manufacturer specifications) were generated for 30 sec. To harvest the virus adsorbed on to the filters, virus-containing filters were immersed in 400 μl of DI water for 5 min, vortexed, and then removed from suspension. The virus suspension was centrifuged at 13,300 rpm at 4°C for 20 min. After completely removing the supernatant from the pellet, 400 μl of PBS was added to dissolve the virus. To harvest the virus that passed through the mask filters, the conical tube was centrifuged at 2000 rpm for 5 min after vortexing, and the contents were transferred to a microcentrifuge tube. The transferred contents were centrifuged at 13,300 rpm at 4°C for 20 min and after removing the supernatant, the contents were resuspended in 400 μl of PBS.

### Filtration efficiency tests

Filter-adsorbed viruses were acquired using the method described above. Viruses in the aerosol were obtained by generating a viral aerosol in a 15 ml conical tube containing 1 ml of DI water, centrifuged at 13,300 rpm at 4°C for 20 min, and then dissolved in 400 μl of PBS. The amount of virus obtained in each step was measured based on protein quantification. Virus concentrations were measured with bicinchoninic acid assay (BCA protein assay kit; Thermo Fischer Scientific, Waltham, MA) with bovine serum albumin (BSA) as a standard. The amount of protein in the samples of aerosolized virus and captured virus on mask filer were determined. The amount of virus passed through the mask filter was calculated as follows:

Theamountofviruspassedthroughthemaskfilter=Theamountofaerosolizedvirus−Theamountofcapturedvirusonmaskfilter

The filtration efficiency was calculated as the ratio of the total amount of virus particles from the filter to the total aerosolized virus:

$$Filtration\;efficiency\;(\%)=\frac{Filtered\;virus}{Total\;aerosolized\;virus}\;x\;100$$

### Pathogenicity in mice

Six-week-old female BALB/c mice were purchased from NARA Biotech (Seoul, South Korea). Mice were randomly divided into groups (n = 10 per group) and intranasally infected with 50 μl of viruses that bypassed the mask filters (passed virus). To measure viral replication in the lungs, five mice from each group were sacrificed 4 days post-infection (4 dpi), and the remaining mice from monitored daily to record changes in body weight and mortality. Mice were humanely euthanized once bodyweight loss exceeded 25% of its initial body weight.

### Influenza virus titer and lung viral titer

Mice were sacrificed 4 dpi to collect whole lung tissues, which were minced using sterilized glass slides and 1 ml of RPMI-1640 media (Lonza, Basel, Switzerland). Lung homogenates were centrifuged at 2000 rpm for 10 min and the supernatants were frozen and kept at -80°C until use. Madin-Darby canine kidney (MDCK) cells were grown in 6 well plates using Dulbecco’s Modified Eagle’s Medium (DMEM) supplemented with 10% fetal bovine serum (FBS) and 1% penicillin/streptomycin at 37°C with 5% CO_2_. To determine the virus titer, ten-fold serially diluted virus samples or lung supernatants were inoculated onto a confluent monolayer of MDCK cells as described previously [12].

### Hemagglutinin activity

To measure influenza hemagglutinin (HA) protein titers, viruses on mask filter were resuspended in 400 μl of PBS. Fifty microliters of virus samples were serially diluted with PBS in a round-bottom 96-well plate and then mixed with 50 μl of 0.5% chicken red blood cells suspension. The 96-well plate was incubated for 1 h at room temperature to determine end-point titers.

### Sodium dihydrogen phosphate efficiency tests

The sodium dihydrogen phosphate efficiency test is an experiment to confirm the effect of salts on viruses. The salt solutions were prepared by dissolving 0.5M, 1M, 2M, 3M, 4M or 5M of sodium dihydrogen phosphate (NaH_2_PO_4_, Sigma Aldrich, St. Louis, MO) or 1M sodium chloride (NaCl, Sigma Aldrich, St. Louis, MO) in DI water with stirring at 400 rpm, 80°C. DI water and PBS were used as control. To investigate the changes in viral activity induced by salts, 10 μl of influenza A/Hong Kong/1/1968 (H3N2) viruses (2 mg/ml, 0.4 mg/ml, and 0.2 mg/ml) and 100 μl of salt solution (0.5M, 1M, 2M, 3M, 4M or 5M of NaH_2_PO_4_) were mixed and incubated at room temperature for 1h. To assess the salt-mediated reduction of viral activity in a time-dependent manner, 10 μl of influenza A/Hong Kong/1/1968 (H3N2) virus (2 mg/ml) and 100 μl of 1M salt solutions (NaH_2_PO_4_ and NaCl) were mixed and incubated at room temperature for 5, 10, 30 or 60 min. These mixtures were centrifuged at 13,300 rpm at 4°C for 20 min and after supernatant removal, the pellets were resuspended in 400 μl of PBS and subsequently used to measure hemagglutinin activity and virus titer.

### Statistics

All parameters were recorded for individuals within all groups and data sets are presented as mean ± SEM. Statistical comparisons of data were carried out by a one-way ANOVA with Tukey’s post hoc test or a Student’s *t*-test using PC-SAS 9.4 (SAS Institute, Cary, NC, USA) A *p*-Value < 0.05 was considered to be significant.

## Results

### Characterization of mask filters coated with salt

**Fig 2:**
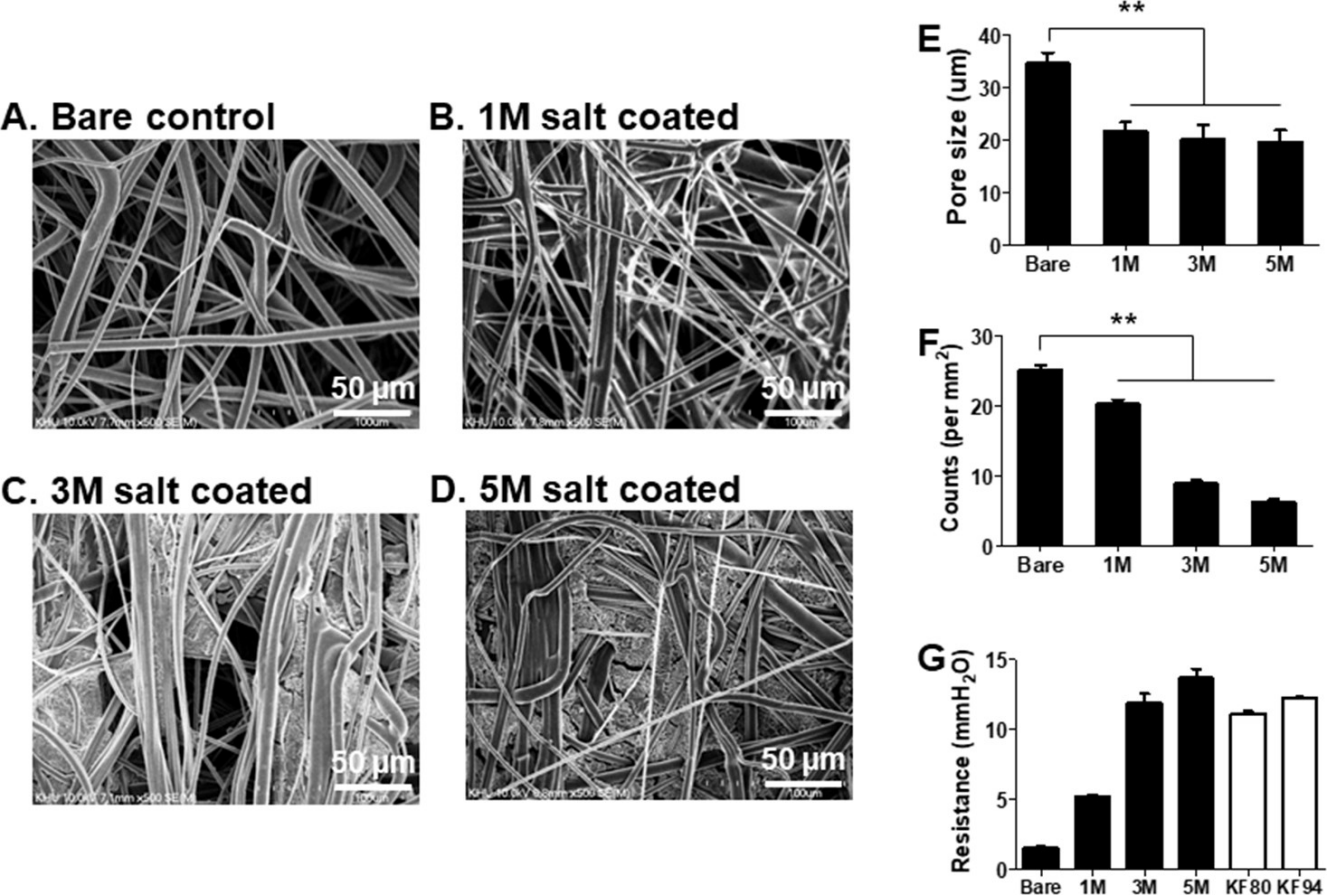
SEM images of filters coated with sodium dihydrogen phosphate. Surgical mask filters were coated with various concentrations of sodium dihydrogen phosphate and the pore size, pore count, and resistance (mmH_2_O) were assessed. A. non-coated bare filter control, B. Filter coated with 1M of coating solution, C: Filter coated with 3M of coating solution, D: Filter coated with 5M of coating solution. E. Pore size of filter (**P < 0.01), F. Pore counts of filters (**P < 0.01). G. Air resistance of filters were tested at non-coated bare control, 1M coated, 3M coated and 5M coated mask filters, of which corresponding filters are abbreviated as Bare, 1M, 3M and 5M, each respectively. KF80 and KF94 are abbreviations for mask filters which are commercially available in Korea.

The experiment was designed as indicated in Fig 1 to assess the virus filtration efficiency of salt-coated mask filters. Captured viruses in the filters were used to measure HA activity and passed viruses were used to assess virus infectivity and pathogenicity in mice. The mask filters were coated with sodium dihydrogen phosphate solutions at different concentrations along with surfactants to improve the wetting properties of the coating solution. Scanning electron microscopy analysis indicated that the pore size and filter pore counts were significantly decreased compared to bare control (Fig 2A–2F). Next, the air resistance of the filters coated with 1M, 3M, or 5M was found to be increasing in a salt concentration-dependent manner. The resistances of commercial mask filters KF80 and KF94 (a functional mask made in Korea to block fine dust, pore size: over 600 μm) used publicly in Korea were found to be similar to those coated with 3M or 5M NaH_2_PO_4_, indicating its potential for human use (Fig 2G).

### Virus HA activity and infectivity were lost in a salt solution

To assess the salt-mediated changes to viral HA activity and infectivity, viruses were incubated under various salt concentrations. As shown in Table 1, when viruses were incubated with sodium dihydrogen phosphate for 1h, salt concentrations and HA titers were found to be inversely proportional. Plaque formations were not detected from viruses (2mg/ml) treated with 3M or 5M sodium dihydrogen phosphate, indicating that all viruses were inactivated. Next, we tested the infectivity of viruses incubated with 1M of salt solution for 5, 10, 30, and 60 mins. As seen in Table 2, sodium dihydrogen phosphate treatment significantly decreased viral HA activity and viral titers at all incubation time points. Incubating the viruses with sodium dihydrogen phosphate for 5 min lessened virus infectivity and by 30 min incubation, 461-fold plaque reductions were observed. More than 99% of plaque reductions were observed at 60 min, indicating that sodium dihydrogen phosphate rapidly inactivated the viruses in a short time. On the contrary, substantial reductions in HA activity or virus infectivity were not observed from sodium chloride-treated viruses. These results indicated that the viral HA activity and infectivity were lost in the sodium dihydrogen phosphate solution, but not in the sodium chloride solution.

**Table 1:** HA activity and infectivity of viruses in various concentrations of sodium dihydrogen phosphate.

Virus Conc. (mg/ml)	Assay	Groups							
		PBS	D.W	0.5M	1M	2M	3M	4M	5M
**2**	**HA titer (/ml)**	**25600±0**	**20480±0**	8533±2956*	6827±2956*	5973±3910*	4267±1478*	2560±0*	1707±739*
	**Virus plaque (x10** ^**5**^ **pfu/ml)**	**113±60**	**-**	**-**	0.0025±0.0005*	**-**	**0**	**-**	**0**
**0.4**	**HA titer (/ml)**	**1920±905**	**1280±0**	533±185*	426±185*	426±185*	373±244*	267±92*	187±122*
	**Virus plaque (x10** ^**5**^ **pfu/ml)**	**10±8**	**-**	**-**	**0**	**-**	**0**	**-**	**0**
**0.2**	**HA titer (/ml)**	**960±452**	**640±0**	266±92*	213±92*	133±46*	106±46*	66±23*	40±0*
	**Virus plaque (x10** ^**3**^ **pfu/ml)**	**1.82±0.9**	**-**	**-**	**0**	**-**	**0**	**-**	**0**

Live influenza viruses (A/H3N2) were incubated in 0.5M, 1M, 3M, 4M, and 5M of sodium dihydrogen phosphate solution for 1h, and HA activity and infectivity of viruses were determined (n = 4, *P < 0.05 vs. PBS or D.W control).

**Table 2:** Salt-mediated changes to viral HA activity and infectivity over time.

Virus Conc. (mg/ml)	Salt solution	Assay	Incubation time			
			5 min	10 min	30 min	60 min
**2**	**PBS control**	**HA titer (/ml)**	**27307±11824**	**27307±11824**	**30630±17659**	**27307±11824**
		**Virus plaque (x10** ^**3**^ **pfu/ml)**	**10467±5193**	**12133±7054**	**10067±5585**	**10133±5586**
	**Sodium chloride (NaCl)**	**HA titer (/ml)**	1**7067±5912**	**13653±5912**	**13653±5912**	**13653±5912**
		**Virus plaque (x10** ^**3**^ **pfu/ml)**	**11267±2833**	**9467±2215**	**9400±3382**	**10866±2670**
	**Sodium dihydrogen phosphate (NaH**_**2**_ **PO**_**4**_ **)**	**HA titer (/ml)**	**13653±5912**	10240±0*	8533±2956*	6827±2956*
		**Virus plaque (x10** ^**3**^ **pfu/ml)**	1600±1083*	279±140*	22.7±16.7*	0.25±0.05*

Live influenza viruses (A/H3N2) were incubated with 1M of sodium dihydrogen phosphate or sodium chloride solutions and the changes in viral properties were determined by hemagglutinin activity assay and influenza plaque assay. Hemagglutinin activities and virus plaque titers were gradually decreased following incubation with sodium dihydrogen phosphate solution, and at 1h, over 99% of viruses lost infectivity (n = 5, *P < 0.05 vs. PBS control).

### Pathogenicity of viruses captured on and passed by filters by aerosolized high amount of viruses

**Fig 3:**
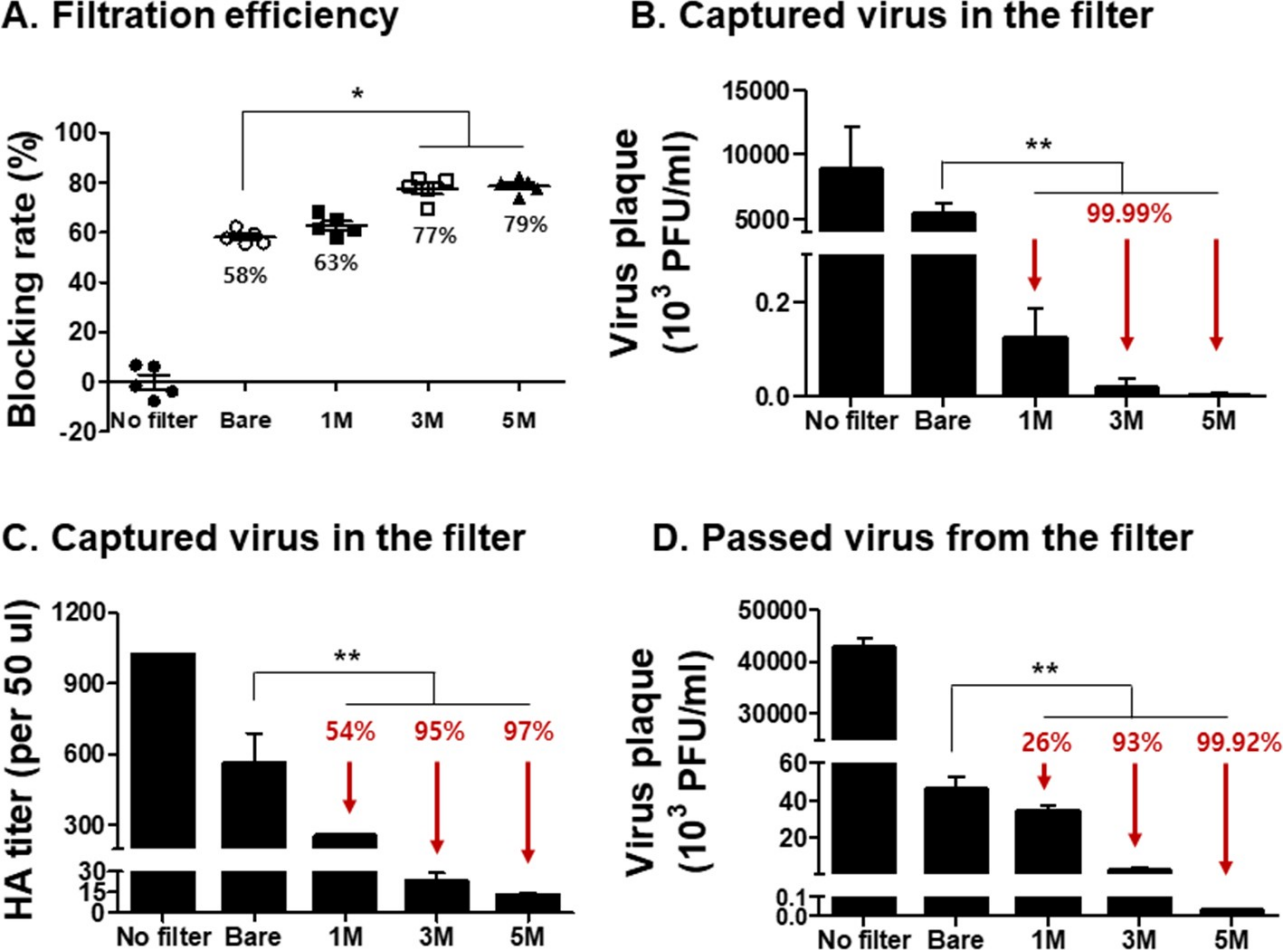
Virus blocking rate, virus plaques, and HA titers from captured or passed viruses using a high amount of aerosolized viruses. Surgical mask filters were coated with 1, 3, and 5M of salt then exposed to a high amount of aerosolized virus (A/H3N2, 8mg/ml, 3.5x10^6^ pfu). (A) Virus block efficiency of salt coated filters (n = 5, mean±SEM, *P < 0.05), (B) virus titer captured in the mask filter (n = 5, mean±SEM, **P < 0.01), (C) HA titer of virus captured in the mask filter (n = 5, mean±SEM, **P < 0.01), and (D) titer of virus passing through the mask filters (n = 4, mean±SEM, **P < 0.01). No filter refers to a virus control group, which does not pass through the mask filters. Bare refers to a control group, where aerosolized viruses pass through uncoated mask filters.

High amounts of influenza virus (A/H3N2, 8 mg/ml, 3.5x10^6^ pfu) was aerosolized and upon exposing the aerosols to the mask filters, virus pathogenicity from both captured and passed viruses were determined. As seen in Fig 3, filter-captured viruses from bare, 1M, 3M, and 5M of NaH_2_PO_4_ were 58%, 63%, 77%, and 79%, each respectively (A). In contrast to the bare filter, almost all of the viruses exposed to the sodium dihydrogen phosphate-coated filters lost their infectivity (B). HA titer reductions were also influenced by increasing sodium dihydrogen phosphate concentrations (1M: 54%, 3M: 95%, 5M: 97%) (Fig 3C). Similar to captured viruses, passed viruses also underwent drastic reductions in viral infectivity following sodium dihydrogen phosphate exposure, with increased salt concentration exerting enhanced viral suppression (1M: 26%, 3M: 93%, and 5M: 99.9%) compared to bare control (Fig 3D).

**Fig 4:**
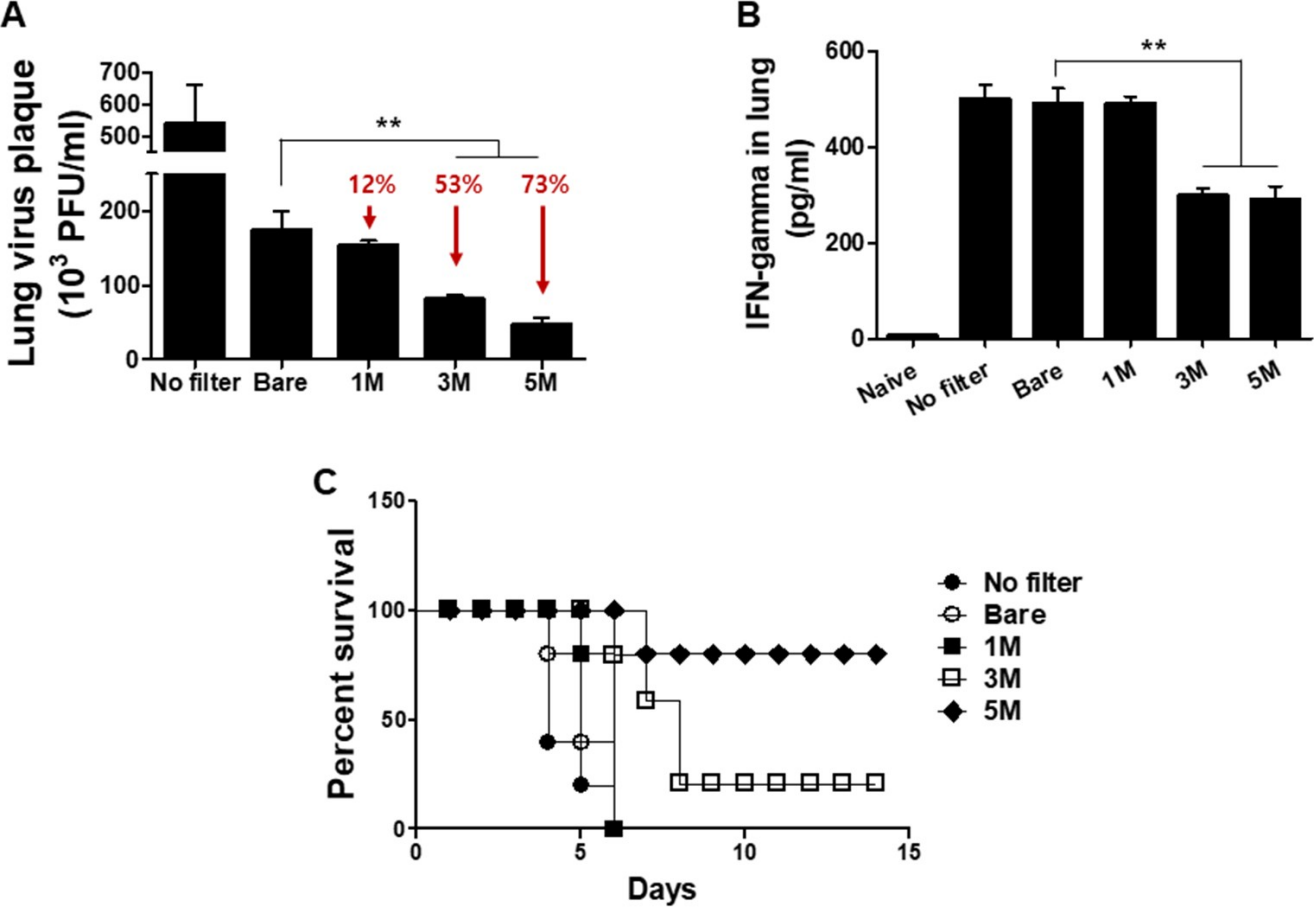
Virus infectivity study in mice using viruses passing through the filters. Mask filters coated with 1, 3, and 5 M of salt and aerosolized influenza viruses (A/H3N2, 8mg/ml, 3.5x10^6^ pfu) were passed through filters. Passed viruses were harvested to infect mice, and virus titers and inflammatory cytokine in the lung, mouse survivals were assessed. (A) Lung virus titers (n = 6, mean±SEM, **P < 0.01), (B) lung inflammatory cytokine (IFN-gamma)) assay (n = 5, mean±SEM, **P < 0.01), and (C) survival rate.

To determine the pathogenicity of the viruses passing through the filter, 50 μl of the passed virus samples were inoculated into mice. Virus replication and production of the inflammatory cytokine IFN-γ were examined from the lungs of mice at 4 dpi. Compared to bare control, viral plaque reductions induced by 3M and 5M salt treatments were 53% and 73%, respectively (Fig 4A). IFN-γ levels were similar between bare control and 1M sodium dihydrogen phosphate-treated groups, whereas significant reductions in IFN-γ were detected from the lungs of mice infected with passed viruses exposed to 3M or 5M sodium dihydrogen phosphate (Fig 4B). Higher survival was observed from mice infected with the passed viruses exposed to 3M (20%) or 5M (80%) salts, while all of the mice infected with the passed viruses collected from 1M salt filter and bare filter perished (Fig 4C). These results indicated that virus infectivity and pathogenicity were decreased or lost at 3 and 5 M of salt concentrations. Although filtration efficiencies were similar between 3M and 5M sodium dihydrogen phosphate-treated filters, discrepancies were observed from passed viruses exposed to the two concentrations with lesser pathogenicity being observed from the latter.

### Virus infectivity and pathogenicity induced by a low amount of viruses H3N2 and H5N1

**Fig 5:**
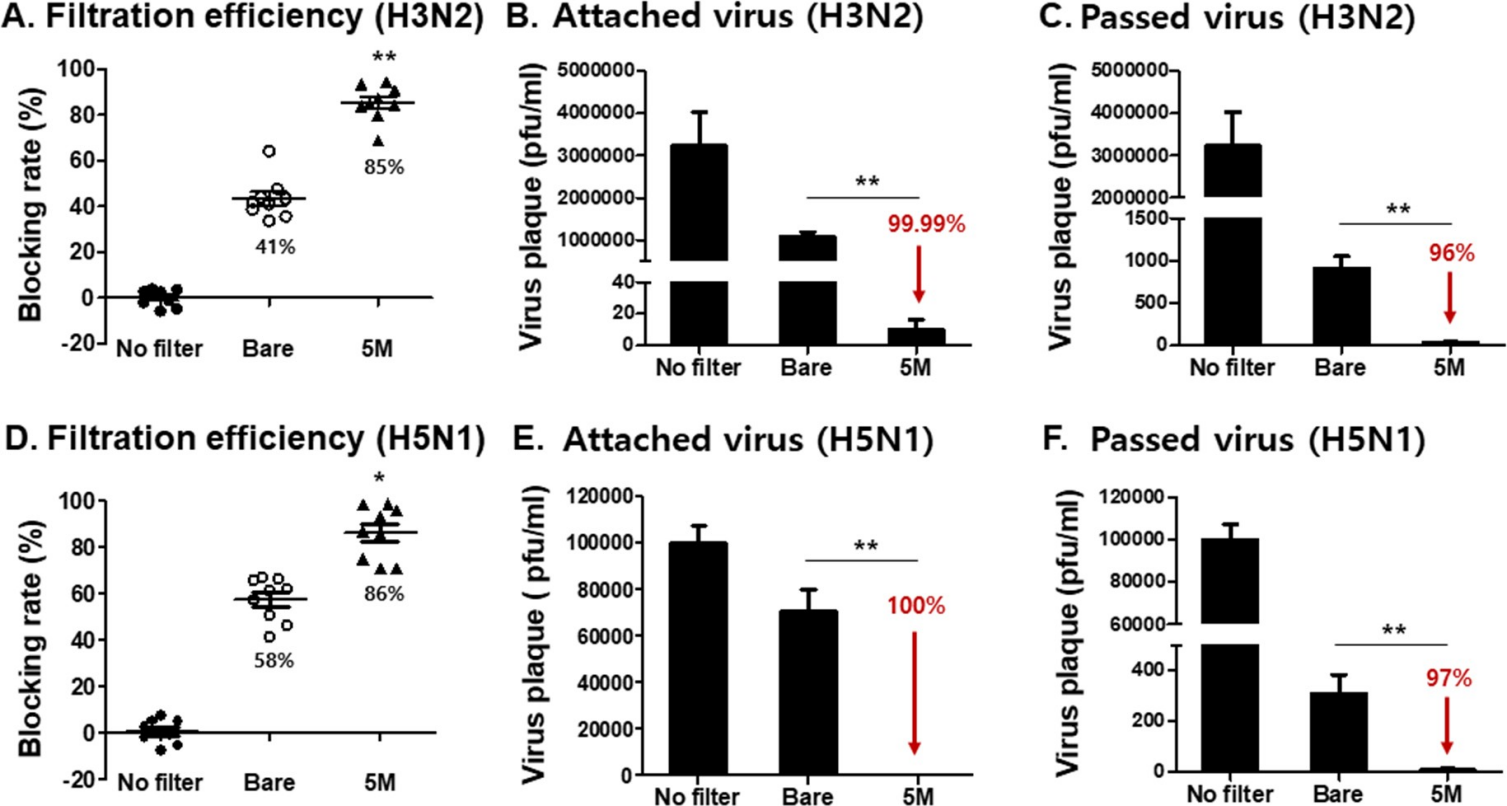
Efficacy of mask filters coated with salt against low amounts of viruses (A/H3N2, A/H5N1). Surgical mask filters were coated with 5M of salt and low amounts of aerosolized virus (A/H3N2, 2 mg/ml, 1.3x10^6^ pfu, A/H5N1, 2 mg/ml, 4x10^4^ pfu) were used. (A) Blocking efficiency of filter coated with 5M of salt against A/H3N2 virus (n = 9, mean±SEM, **P < 0.01), (B) Virus plaque titers of H3N2 virus captured on the mask filter (n = 5, mean±SEM, **P < 0.01), and (C) Virus titers of H3N2 virus passing through the mask filters (n = 5, mean±SEM, **P < 0.01). (D) Blocking efficiency of salt-coated mask filters against A/H5N1 virus (n = 9, mean±SEM, *P < 0.05), and (E) Virus titers of H5N1 captured in the mask filter (n = 5, mean±SEM, **P < 0.01), (F) Virus titer of H5N1 passing through the mask filters (n = 5, mean±SEM, **P < 0.01).

**Fig 6:**
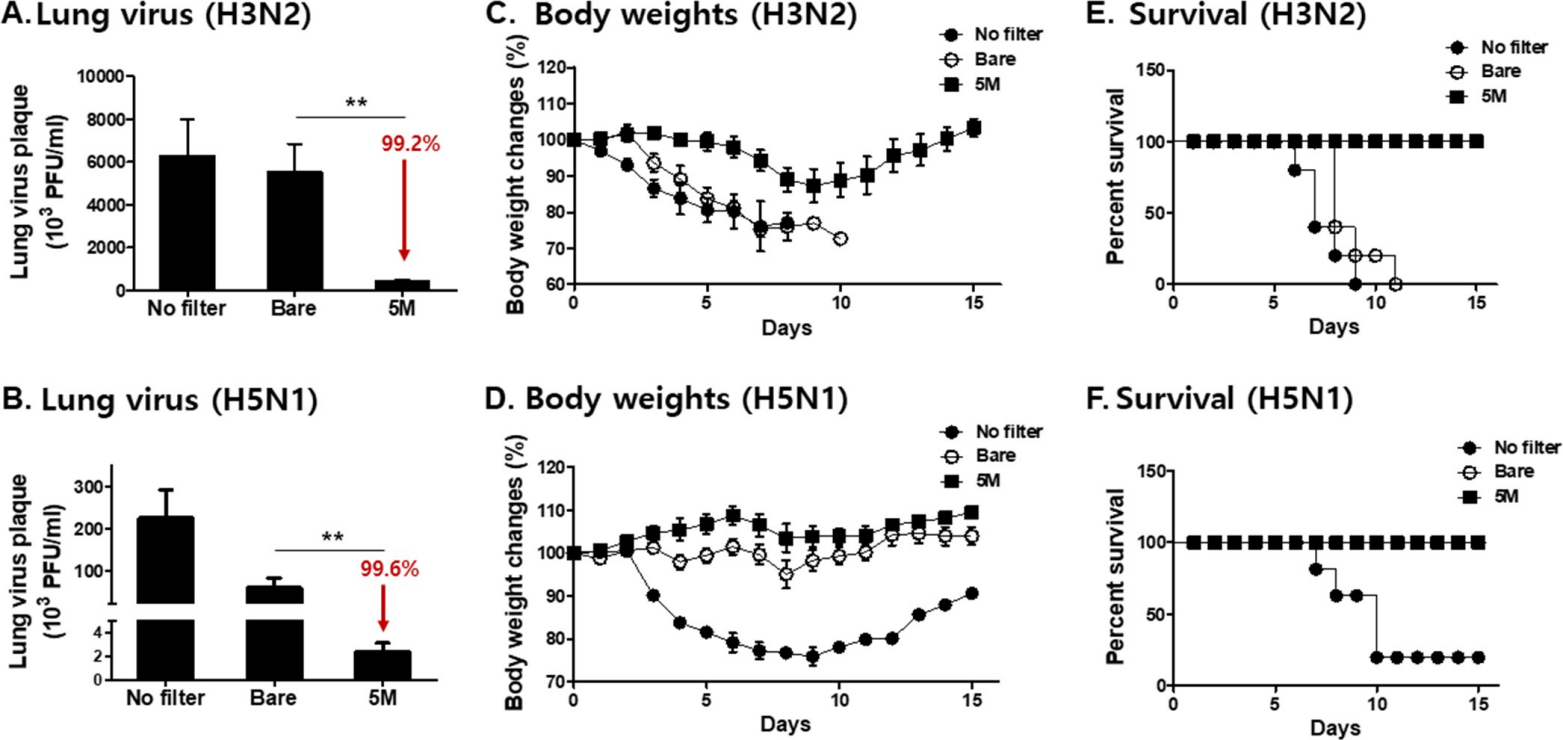
Virus infectivity study in mice using viruses passing through the filters. Mask filters coated with 5 M of salt and aerosolized influenza viruses (A/H3N2, 2mg/ml, A/H5N1, 2mg/ml) were passed through the filters. Passed viruses were harvested to infect mice, and virus titers in the lung, bodyweight changes, and survival were assessed. Lung virus titers (A and B, n = 6, mean±SEM, **P < 0.01), bodyweight changes (C and D, n = 5), and survival rates (E and F, n = 5).

To confirm the reproducibility of this experiment, virus infectivity and pathogenicity from captured and passed viruses were tested using low concentrations of viruses. Aerosolized A/H3N2 and A/H5N1 viruses (1.3x10^6^ pfu and 4x10^4^ pfu, respectively) were exposed to 5M salt-coated mask filters, with filtration efficiencies for H3N2 and H5N1 in salt-coated and bare filters being 85%, 86%, 41%, and 58%, each respectively (Fig 5A and 5D). Exposing aerosolized viruses to 5M sodium dihydrogen phosphate resulted in diminished plaque formations from both captured and passed viruses, regardless of the virus strain (Fig 5B, 5C, 5E and 5F). The virus samples (A/H3N2 and A/H5N1) passing through the filter were collected for mouse infection. Similar to above, regardless of the strain used, pulmonary viral replication for the viruses that bypassed the 5M sodium dihydrogen phosphate-coated filters was significantly less than those bypassing the bare filter (Fig 6A and 6B; 99% reduction compared to bare control for both strains). Aerosolized and bare filter-passing viruses of the H3N2 strain caused 100% mortality in mice, whereas all of the mice infected with the viruses passing through the 5M salt-coated filters survived (Fig 6C and 6E). Mice infected with the H5N1 viruses from 5M salt-coated filters showed no bodyweight changes (Fig 6D) and all mice survived, while the bare control group lost 5% of its initial bodyweight post-infection (Fig 6F). These results indicated that virus infectivity and pathogenicity were decreased or lost upon contact with 5M sodium dihydrogen phosphate.

## Discussion

With the ongoing COVID-19 pandemic, face masks have become a necessity and are being widely used by the general population. However, even with the mask-wearing practice, millions of people have been infected and over eighty hundred and thousand deaths occurred worldwide [13–16]. More than 150,000 healthcare workers have been infected by the COVID-19 virus according to data from just nine countries–Denmark, Germany, Hungary, Ireland, Italy, Russia, Spain, Turkey, and Ukraine [17]. To limit the spread of the disease, the effectiveness of face masks needs to be studied more extensively. Our results indicated that aerosolized viruses can penetrate the mask filter, with 58% of H3N2 viruses being blocked under high amounts of aerosolized viral particle conditions. Filtration efficacies for low amounts of aerosolized viral particles for H3N2 and H5N1 were 41% and 58%, respectively. Our findings are consistent with the clinical trial outcomes, which reported mask-equipping healthcare personnel being infected with respiratory viruses [9]. Thus, we concluded that mask filter modification is needed and tested the efficacy of mask filters coated with sodium dihydrogen phosphate.

Viruses exposed to humans in the air could be at a high or low concentration. Thus, mask filters coated with various concentrations of salts were exposed to the aerosolized viruses at high and low amount conditions. Exposing mask filters coated with 3M or 5 M of salt to a high amount of viruses resulted in a 99% viral infectivity reduction from captured viruses, which is consistent with the HA titers being reduced by 95% and 97% for salt-coatings of 3M and 5M, respectively. In passed viruses, 3M and 5M sodium dihydrogen phosphate coating reduced the viral infectivity by 93% and 99%, respectively. Similar results were observed from mask filters exposed to a low amount of aerosolized viruses. Passed viruses collected from the low concentration of aerosolized viruses were used for pathogenicity study in mice. Lung virus titer results revealed that compared to bare control or 1M sodium dihydrogen phosphate-coated filters, passed viruses from 3M and 5M salt-coated filters demonstrated less viral infectivity in murine lungs. The lowest pathogenicity was observed from passed viruses collected from 5M sodium dihydrogen phosphate-coated filters. These results indicated that viruses aerosolized at both high and low concentrations can be effectively killed upon contact with sodium dihydrogen phosphate-coated mask filters.

Our findings indicate that incubating the influenza virus (A/H3N2) for 1 h with sodium dihydrogen phosphate solutions can result in a near-complete killing of the virus, with increasing salt concentration exerting a stronger effect on HA activity reduction. Incubating the virus with the salt solution for 5 min resulted in 28-fold viral infectivity reduction compared to PBS control, and prolonged exposure to 1M sodium dihydrogen phosphate solution further inhibited viral infectivity. Interestingly, significant reductions in virus HA activity or viral infectivity were not observed following sodium chloride treatment in the current study. Since sodium chloride crystallization has been reported to induce virus structural damage [10], sodium dihydrogen phosphate may have an additional property that contributes to lessening the HA activity and virus inactivity. Sodium dihydrogen phosphate solution is weakly acidic (pH 4.0) and it was reported that excessive osmotic stress in such an environment can trigger damages to the virus morphology and function [18,19], which may have contributed to the HA activity and infectivity reductions in the present study. The inclusion of phosphonates in drugs has been reported to inhibit influenza virus infection [20–22]. Since phosphates are cheap, odorless, and tasteless, their application as a mask filter coating would have a great impact. Mask filter modification strategy could solve the problems faced by commercially available masks, such as low virus blocking rate, secondary infection, and mask filter contamination during use and disposal [23,24].

## Conclusions

Mask filter modification induced by sodium dihydrogen phosphate coating provided high virus filtration efficiency. Over 95% of the influenza viruses (A/H3N2, A/H5N1) collected from both filter-captured and filter-passing portions lost their HA activity and infectivity *in vitro* and *in vivo*. The current findings would greatly benefit public health by providing highly efficient mask filters that prevent respiratory virus transmission.
